# Omentin-1 Ameliorated Free Fatty Acid-Induced Impairment in Proliferation, Migration, and Inflammatory States of HUVECs

**DOI:** 10.1155/2020/3054379

**Published:** 2020-03-27

**Authors:** Yubin Chen, Fen Liu, Fei Han, Lizhi Lv, Can-e Tang, Fanyan Luo

**Affiliations:** ^1^Department of Cardiac Surgery, Xiangya Hospital, Central South University, Changsha 410008, Hunan, China; ^2^The Institute of Medical Science Research, Xiangya Hospital, Central South University, Changsha 410008, Hunan, China

## Abstract

**Objectives:**

Endothelial cell injury is a critical pathological change during the development of atherosclerosis. Here, we explored the effect of omentin-1 on free fatty acid- (FFA-) induced endothelial cell injury.

**Methods:**

An FFA-induced endothelial cell injury model was established to investigate the role of omentin-1 in this process. Cell proliferation was analyzed with the Cell Counting Kit assay and flow cytometry. Scratch and transwell assays were used to evaluate cell migration. Factors secreted by endothelial cells after injury were detected by western blotting, reverse-transcription quantitative polymerase chain reaction, and cellular fluorescence assay.

**Results:**

Omentin-1 rescued the FFA-induced impaired proliferation and migration capabilities of human umbilical vein endothelial cells (HUVECs). It decreased the number of THP-1 cells attached to HUVECs in response to injury and inhibited the FFA-induced proinflammatory state of HUVECs.

**Conclusion:**

Omentin-1 could partly ameliorate FFA-induced endothelial cell injury.

## 1. Introduction

The vascular endothelium is a continuous single-cell lining of the circulatory system that forms an interface between the blood and the vascular wall. This layer possesses multiple functions, including maintenance of thrombohemorrhagic balance and regulation of inflammatory response [1]. Some pathological conditions such as dyslipidemia and hyperglycemia may induce endothelial cell injury, which is characterized with increased cell apoptosis, decreased cell proliferation, and activation of proinflammatory signaling pathways [2, 3]. Such injuries interrupt the homeostasis of the vascular endothelium and cause inflammatory responses, thereby contributing to the early stage of coronary heart disease (CHD) [4].

The coronary artery is surrounded by the epicardial adipose tissue (EAT), a special visceral adipose tissue without fascia separation. The volume of the EAT is positively associated with CHD, independent of blood pressure, low-density lipoprotein cholesterol, high-density lipoprotein cholesterol, and some other traditional risk factors [5]. The adipose tissues, including EAT, secrete high levels of nonesterified fatty acids during lipolysis [6, 7]. These free fatty acids (FFAs) activate Toll-like receptor (TLR)-4, eventually leading to an increase in nuclear factor kappa B (NF-*κ*B) activity. This changes the profile of endothelial cells toward an inflammatory state [8]. FFAs could also downregulate the synthesis of vascular endothelial growth factor (VEGF) and inhibit the repair of endothelial wounds [9]. Furthermore, excessive secretion of FFAs impairs the cardiac structure [10]. FFAs may also contribute to the onset and development of CHD.

Omentin-1 is a novel adipocytokine expressed in the EAT, omental adipose tissue, and other visceral adipose tissue depots [11]. It inhibits the tumor necrosis factor-*α* (TNF-*α*)-induced endothelial cell inflammatory state [12], downregulates the extracellular matrix synthesis of vascular smooth muscle cells [13], and attenuates arterial calcification in osteoprotegerin-deficient mice [14]. Despite these vascular protective effects, omentin-1 is known to predict coronary collateral circulation [15]. Further, the plasma level of omentin-1 is associated with CHD and heart failure in senile patients [16]. These findings suggest that omentin-1 may exert antiatherosclerosis effects. However, the role of omentin-1 in FFA-induced endothelial cell injury is still unknown. Here, a FFA-induced endothelial cell injury model was established to explore the effect of omentin-1 on this process.

## 2. Materials and Methods

### 2.1. Cell Culture and Treatment

Human umbilical vein endothelial cells (HUVECs) were obtained from Procell (CL-0122) and cultured in Dulbecco's modified Eagle's medium (DMEM, HyClone) supplemented with 10% fetal bovine serum (FBS) and 1% penicillin-streptomycin (P/S). Cells from passages 3 to 8 were used for experiments. Palmitic acid (PA; P5585, Sigma), a representative FFA in vivo, was prepared in dimethyl sulfoxide (DMSO). Confluent HUVECs were serum-starved for overnight in non-FBS DMEM, and then the medium was replaced with FBS-containing DMEM. HUVECs were incubated with PA and/or recombinant human omentin-1 (9137-IN-050, R&D) as follows: control (0 mM PA in phosphate-buffered saline [PBS]), DMSO (0 mM PA in DMSO), 1 mM PA, 1 mM PA + 100 ng/mL omentin-1, 1 mM PA + 150 ng/mL omentin-1, or 1 mM PA + 200 ng/mL omentin-1 for 24 h.

THP-1 cells were purchased from Procell (CL-0233) and cultured in Roswell Park Memorial Institute (RPMI)-1640 (HyClone) medium supplemented with 15% FBS and 1% P/S. Cells from passages 3 to 5 were used for experiments.

### 2.2. Cell Proliferation Assay

Serum-starved cells (1 × 10^3^) were seeded into 96-well plates and treated with PA and/or recombinant human omentin-1 (concentration as mentioned above) for 24 h. Cell proliferation was measured using the Cell Counting Kit-8 (CCK-8) (40203ES76, Yeasen) assay according to the manufacturer's recommendation.

### 2.3. Cell Cycle Analysis

Cell Cycle Analysis Kit (FXP021, 4A BIOTECH) was used to analyze changes in cell cycle phases following treatment of HUVECs with PA and/or recombinant human omentin-1 for 24 h. The cells were collected after treatment, washed thrice with PBS, and fixed in 95% ethanol at 4°C overnight. Cells (1 × 10^6^) were stained with propidium iodide (PI) and examined using flow cytometry. Data were analyzed using FlowJo 7.6.1 software.

### 2.4. Scratch Assay

HUVECs were seeded into six-well plates at 2 × 10^5^ cells/well and serum-starved for 24 h. The cell layer on the surface of each well was gently scratched using 10-*μ*L pipette tips. Wells were rinsed thrice with PBS to remove any cell debris and filled with the medium containing 2% FBS with PA and/or recombinant human omentin-1 (concentration as mentioned above). Images were obtained at 0, 12, and 24 h using a microscope. Wound closure rate ((initial wound area - wound area at 24 h)/initial wound area) was analyzed and calculated using Image Pro Plus 6.0 software.

### 2.5. Migration Assay

The migration of HUVECs was investigated using a 24-well modified Boyden chamber (8 *μ*m, Corning). After treatment with PA and/or recombinant human omentin-1 (concentration as mentioned above) for 24 h, HUVECs were collected and resuspended in serum-free DMEM. Approximately 5 × 10^4^ cells in 200 *μ*L of serum-free DMEM were seeded in the upper chamber, while the lower chamber was filled with 600 *μ*L DMEM containing 10% FBS. After 24 h incubation, the cells on the upper face of the membrane were wiped, while those that migrated to the lower face of the membrane were fixed with 4% paraformaldehyde for 20 min and stained with crystal violet solution (C0121, Beyotime) for 5 min. Images were obtained using a microscope (Leica DM5000 B). The number of migrated HUVECs was counted in three random fields (200× magnification).

### 2.6. Western Blotting Analysis

After treatment for 24 h, HUVECs were lysed using radioimmunoprecipitation assay (RIPA) buffer (WB3100, NCM Biotech) containing 1% phenylmethylsulphonyl fluoride (PMSF G2008, Servicebio). The cell lysis solution was further dissociated with ultrasound treatment and centrifuged at 12000 *×* g at 4°C for 30 min to obtain supernatant. Protein concentration in the supernatant was determined with the bicinchoninic acid (BCA) assay (23227, ThermoFisher Scientific). Proteins (20 *μ*g) were separated on 10% Bis-Tris gel, and the separated bands were transferred to a polyvinylidene fluoride (PVDF) membrane (Millipore IPVH00010). The membrane was blocked in 5% defatted milk at 25°C for 2 h and incubated overnight at 4°C with primary antibodies against the following proteins: tubulin (1 : 4000 dilution; ab6046, Abcam); glyceraldehyde 3-phosphate dehydrogenase (GAPDH) (1 : 4000 dilution; T0004, Affinity); intercellular adhesion molecule-1 (ICAM-1) (1 : 800 dilution; 4915S, CST); monocyte chemoattractant protein-1 (MCP-1) (1 : 1000 dilution; DF7577, Affinity); NF-*κ*B (1 : 1000 dilution; #4764, CST); and NF-kappa-B inhibitor alpha (I*κ*B*α*) (1 : 1000 dilution; #4812, CST). The PVDF membrane was then probed with corresponding secondary antibodies conjugated to horseradish peroxidase (HRP; 1 : 5000 dilution; #S0001, #S0002, Affinity) at 25°C for 70 min and observed using enhanced chemiluminescence (p10100, NCM Biotech) with ChemiDoc XRS Plus (Bio-Rad). The relative protein expression level was analyzed using Image Lab 3.0 software.

### 2.7. Reverse-Transcription Quantitative Polymerase Chain Reaction (RT-qPCR)

Total RNA from HUVECs was extracted using TRIzol reagent (15596018, Invitrogen) and reverse-transcribed with HiScript II QRT SuperMix (R223-01, Vazyme). RT-qPCR was performed on a ViiA 7 system (Applied Biosystems) using the All-in-One qPCR Mix (GeneCopoeia) for 40 cycles to detect the mRNA expression levels of *ICAM-1*, *MCP-1*, *interlukin-1* (*IL-1*), *interlukin-6* (*IL-6*), and *TNF-α*. GAPDH was used for normalization. The primers used in this research are shown in Supplementary [Supplementary-material supplementary-material-1].

### 2.8. THP-1 Cell Adhesion Analysis

Starved HUVECs (1 × 10^5^) were seeded into 12-well plates and treated with PA and/or recombinant human omentin-1 (concentration as mentioned above) for 24 h. The medium was replaced. THP-1 cells were incubated with 10 *μ*M of 2′,7′-bis-(2-carboxyethyl)-5-(and-6)-carboxyfluorescein, acetoxymethyl ester (BCECF-AM) fluorescent probe for 30 min in the dark. Cells were collected, washed thrice with PBS, and resuspended in PBS. 100 μL of THP-1 cell suspension was added to the wells of a 12-well plate containing HUVECs, and the cells were cocultured for 1 h. The medium was discarded, and each well was gently washed thrice with PBS. Images were acquired using a fluorescence microscope (Leica DM5000B). The number of THP-1 cells attached to HUVECs was analyzed using Image Pro Plus 6.0 software.

### 2.9. Immunofluorescence of HUVECs

Starved HUVECs (5 × 10^4^) were seeded into 24-well plates and treated with PA and/or recombinant human omentin-1 (concentration as mentioned above) for 24 h. The medium was discarded, and the cells were rinsed thrice with PBS. The cells were fixed with 4% paraformaldehyde for 20 min, permeated with 0.5% Triton-X100 for 15 min, blocked with normal goat serum for 60 min, and incubated overnight at 4°C with primary antibodies against the following proteins: ICAM-1 (1 : 200 dilution; 4915S, CST); MCP-1 (1 : 200 dilution; GB11199, Servicebio); and NF-κB (1 : 200 dilution; #4764, CST). The cells were then incubated with the corresponding secondary antibody Alexa Fluor 488 goat anti rabbit IgG H&L (1 : 500 dilution; ab150077, Abcam) for 40 min at 37°C. The cells were stained for 5 min with 4′,6-diamidino-2-phenylindole (DAPI; 1 : 1000 dilution; 564907, BD Pharmingen), and images were acquired using a fluorescence microscope (Leica DM5000B). Fluorescence intensity was analyzed using Image Pro Plus 6.0 software.

### 2.10. Statistical Analysis

Data are presented as the means ± SEM. Data were compared using the unpaired Student's *t*-test and one-way analysis of variance (ANOVA) followed by the least significant difference (LSD) post hoc test. Statistical analyses were performed using SPSS 19 software. A value of *P* of <0.05 was considered statistically significant.

## 3. Results

### 3.1. Omentin-1 Reversed the PA-Induced Impairment in the Proliferation and Migration of HUVECs

**Figure 1:**
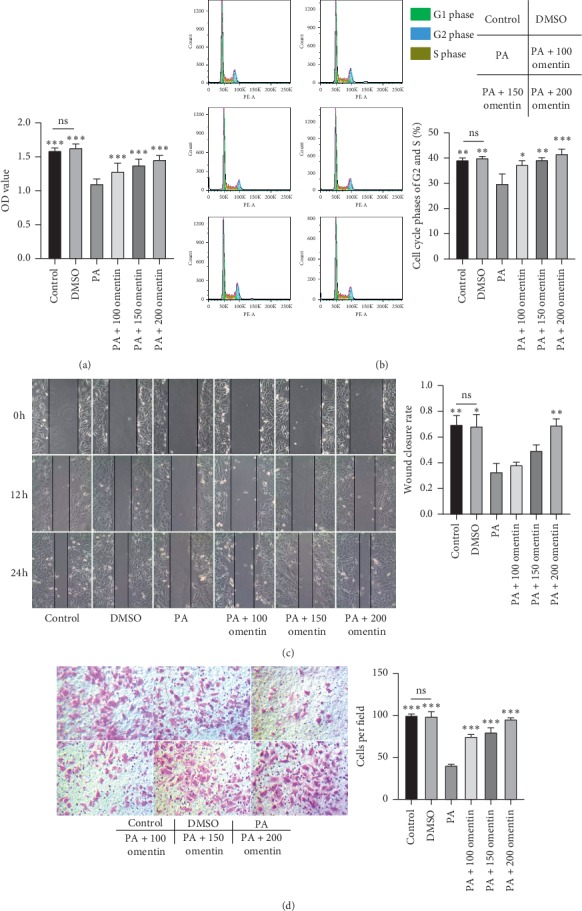
Omentin-1 reversed the PA-induced impairment in the proliferation and migration of HUVECs. (a) Proliferation ability of HUVECs was determined using CCK-8 assay following different treatments. (b) Representative flow cytometry results of cell cycle analysis and quantitative analysis. (c) Representative images of scratch assay (100x magnification) and quantitative analysis of wound closure rate demonstrated the migration capability of HUVECs under different treatments. (d) Representative images of transwell assay (200x magnification) and the number of migrated HUVECs. The values are mean ± SEM of three independent experiments. ^*∗*^*P* < 0.05 vs. PA group, ^*∗∗*^*P* < 0.001 vs. PA group, and ^*∗∗∗*^*P* < 0.0001 vs. PA group.

The treatment of HUVECs with PA alone resulted in the inhibition of their proliferation ability as compared with the control and DMSO treatment (Figure 1(a)). The simultaneous treatment with PA and omentin-1 partly ameliorated the impaired proliferation ability of these cells (Figure 1(a)). Flow cytometry results showed that the proportion of cells in S and G2 phases after treatment with PA alone was lower than that observed after control and DMSO treatment (Figure 1(b)). Thus, HUVEC division was inhibited by PA. The treatment with omentin-1 resulted in the upregulation in S and G2 phases cells as compared with PA treatment, indicating that omentin-1 reversed the PA-induced inhibition of cell division (Figure 1(b)). The scratch and transwell assays demonstrated the impairment in the migration ability of HUVECs by PA treatment and that the cotreatment with omentin-1 could alleviate this effect (Figures 1(c) and 1(d)). These results suggest that PA induces endothelial injury and omentin-1 ameliorates the damage.

### 3.2. Omentin-1 Reduced the PA-Induced Attachment of THP-1 Cells to HUVECs

**Figure 2:**
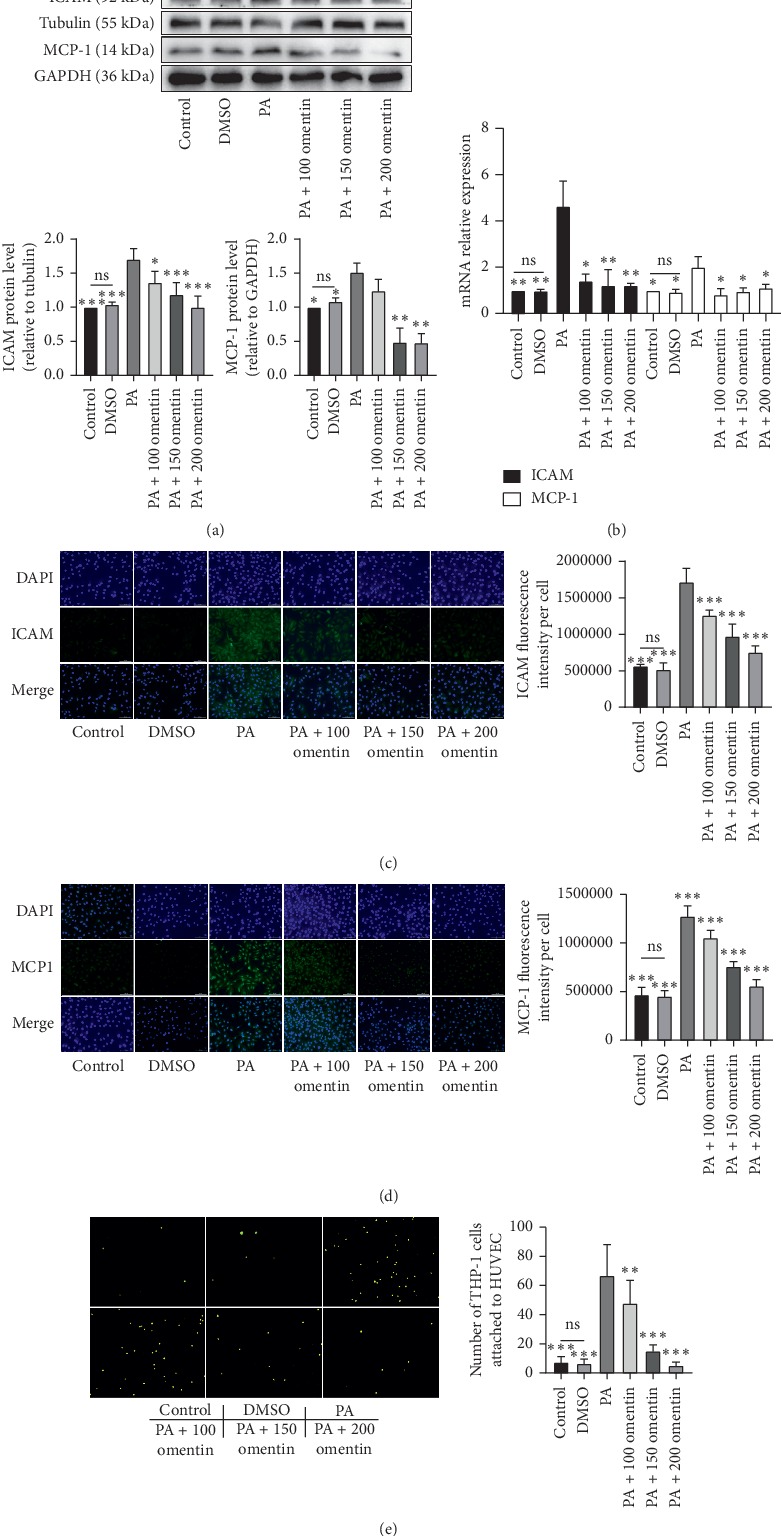
Omentin-1 reduced the PA-induced attachment of THP-1 cells to HUVECs. ICAM-1 and MCP-1 protein expression levels in HUVECs from different groups were detected with western blotting (a) using tubulin or GAPDH as loading control. Quantitative analysis of protein expression is shown as bar graphs. The mRNA expression levels of *ICAM-1* and *MCP-1* were determined with RT-qPCR (b). (c, d) Representative images of cellular fluorescence assay for ICAM-1 and MCP-1 (400x magnification) and the quantitative analysis of fluorescence intensity per cell. (e) The representative pictures of BCECF-AM-labeled THP-1 cells attached to HUVECs in different treatments groups are shown (400x magnification); the number of fluorescent cells was counted. The values are mean ± SEM of three independent experiments. ^*∗*^*P* < 0.05 vs. PA group, ^*∗∗*^*P* < 0.001 vs. PA group, and ^*∗∗∗*^*P* < 0.0001 vs. PA group.

Western blotting analysis showed that the protein expression levels of ICAM-1, an atherosclerosis-associated endothelial-leukocyte adhesion molecule, and MCP-1, an important chemotactic factor, were significantly higher in PA-treated HUVECs than in the control and DMSO treated HUVECs (Figure 2(a)). Omentin-1 inhibited the PA-induced upregulation of ICAM-1 and MCP-1 expression (Figure 2(a)). The PA-induced upregulation in the mRNA expression levels of *ICAM-1* and *MCP-1* was also inhibited following the simultaneous treatment with omentin-1 (Figure 2(b)). Cellular immunofluorescence results further confirmed these findings (Figures 2(c) and 2(d)). The number of THP-1 cells attached to HUVECs significantly increased after treatment with PA alone as compared with that observed in the control and DMSO treatment (Figure 2(e)). However, the cotreatment with omentin-1 reduced the PA-induced increase in the attachment of THP-1 cells to HUVECs (Figure 2(e)). These findings indicate that omentin-1 could prevent the PA-induced adhesion of THP-1 cells to HUVECs and that the role of omentin-1 in the inhibition of the upregulation of PA-induced ICAM-1, and MCP-1 expression, at least in part, contributed to this preventive effect.

### 3.3. Omentin-1 Inhibited the PA-Induced Inflammatory State of HUVECs

**Figure 3:**
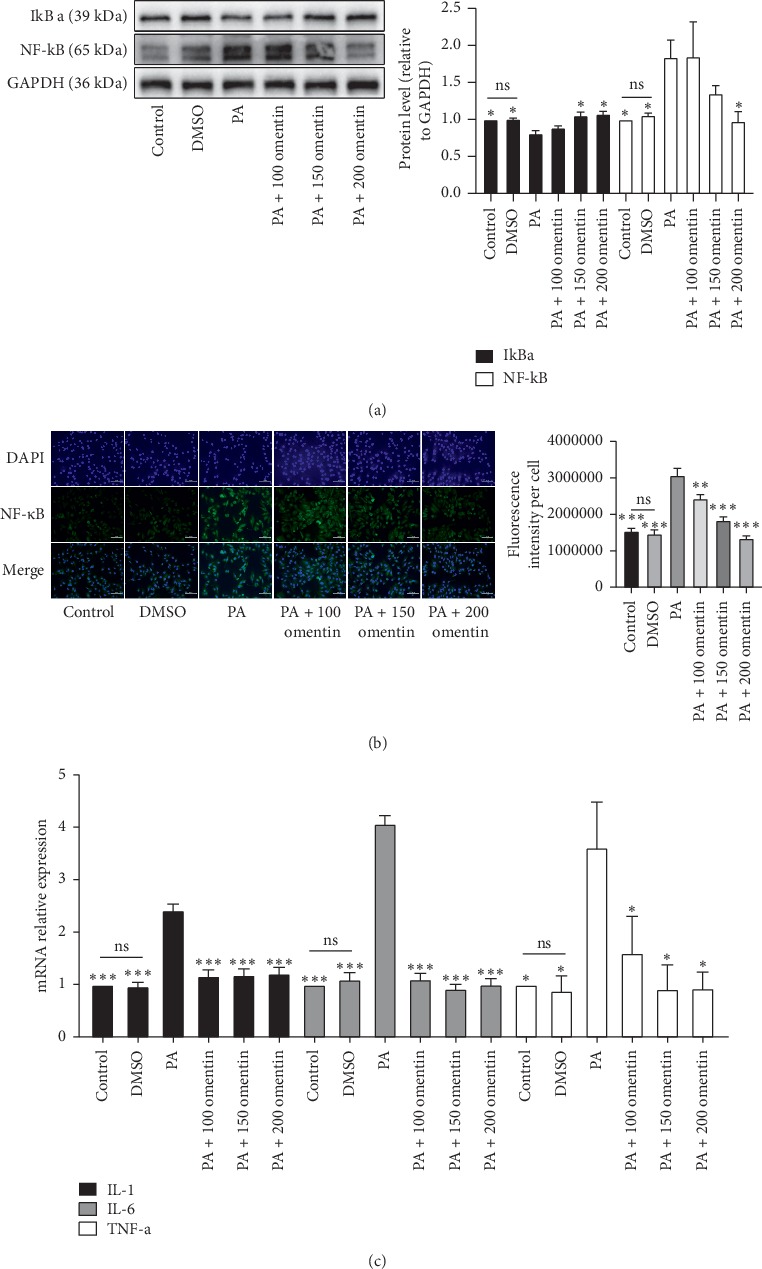
Omentin-1 inhibited the PA-induced inflammatory state of HUVEC. I*κ*B*α* and NF-*κ*B protein expression levels in HUVECs from different groups were detected with western blotting (a) using tubulin as loading control. Quantitative analysis of proteins expression is shown in the right bar graph. (b) Representative images of cellular fluorescence assay for NF-*κ*B (400x magnification) and the quantitative analysis of fluorescence intensity per cell. (c) The mRNA expression levels of *IL-1*, *IL-6*, and *TNF-α* in HUVECs from different treatment groups using RT-qPCR. The values are mean ± SEM of three independent experiments. ^*∗*^*P* < 0.05 vs. PA group, ^*∗∗*^*P* < 0.001 vs. PA group, and ^*∗∗∗*^*P* < 0.0001 vs. PA group.

Both ICAM-1 and MCP-1 are target genes of NF-*κ*B; hence, we detected the protein expression level of NF-*κ*B by western blotting. As a result, we found that the PA-induced upregulation in the expression of NF-*κ*B was ameliorated by omentin-1 cotreatment (Figure 3(a)), and the PA-induced degradation of I*κ*B*α* was prevented by omentin-1 (Figure 3(a)). Thus, omentin-1 inhibited the activation of NF-*κ*B. To further investigate the effect of omentin-1 on the expression of NF-*κ*B, a cellular immunofluorescence assay was performed to detect the expression of NF-*κ*B. The results showed that the fluorescence intensity for NF-*κ*B expression in the cells from PA group was significantly higher than that reported for the control and DMSO treatment groups. On the contrary, omentin-1 reduced the PA-induced change in the fluorescence intensity for NF-*κ*B (Figure 3(b)). The PA-induced upregulation in the mRNA expression levels of *IL-1*, *IL-6*, and *TNF-α*, the target genes of NF-*κ*B, was also ameliorated by omentin-1 (Figure 3(c)). Together these results demonstrate that omentin-1 could partly relieve the PA-induced proinflammatory state of HUVECs by preventing the activation of NF-*κ*B.

## 4. Discussion

CHD, one of the most serious clinical manifestations of atherosclerosis, has become a burden on the global health industry [17]. As a chronic arterial disease, atherosclerosis is characterized with the appearance of fatty steak, development of atheroma, and formation of plaque. The plaque itself or upon its rupture may result in thrombosis and occlusion of the arteries, resulting in the induction of hypoperfusion and damage of the related organ [18]. Endothelial cells play important roles in the cardiovascular system in the maintenance of the vascular tone and regulation of inflammation and thrombosis [19]. Pathological stimuli such as diabetes mellitus, hypertension, and dyslipidemia may provoke endothelial cell injury, characterized with an alteration in the normal functions [20]. To date, endothelial cell injury is one of the earliest pathological changes that can be detected during the development of atherosclerosis [4]. A recent study reported the ability of FFAs to induce endothelial cell injury *in vitro* [21]. In the present study, a model of PA-induced endothelial cell injury was established to determine the role of omentin-1 in this process.

Omentin-1 is a novel adipocytokine abundantly expressed in the EAT, omental adipose tissue, and some other organs [11]. Although the specific receptor of omentin-1 has not been identified, the role of omentin-1 has been extensively explored. Omentin-1 mediated cardiovascular protective effects [22], modulated the functions of insulin [23], and regulated bone metabolism [14]. It was recently reported that the circulating level and mRNA expression level of *omentin-1* in the EAT were significantly downregulated in patients with coronary artery disease (CAD) as compared with those in patients without CAD. In addition, the protein and mRNA expression levels of omentin-1 were lower in the EAT surrounding coronary stenotic segments compared with those in the EAT adjacent to nonstenotic segments, indicating that omentin-1 may be an antiatherosclerosis adipocytokine [24]. However, its role in PA-induced endothelial cell injury is unclear.

The vascular endothelium comprises a continuous monolayer of endothelial cells and regulates coagulation and inflammatory reactions [1]. The integrity of the monolayer of the endothelial cells is important for maintaining these functions [24]. In the present study, the proliferation and migration capabilities of HUVECs were inhibited by PA treatment. These capabilities, which could boost the repair of the single-cell lining, are crucial for the integrity of the vascular endothelium. In comparison with PA treatment, the cotreatment with omentin-1 increased the proportion of S and G2 phase cells, thereby stimulating HUVEC division. Omentin-1 also rescued the PA-induced impaired migration ability of HUVECs. These findings suggest that omentin-1 may contribute to the maintenance of the integrity of the vascular endothelium. The expression levels of ICAM-1 and MCP-1 were also upregulated following exposure of HUVECs to PA alone. As an endothelial-leukocyte adhesion molecule, ICAM-1 expression level increases in atherosclerosis lesions [25]. MCP-1 could recruit and accumulate monocytes in the lesion and accelerate the development of atherosclerosis [26]. The upregulation in the expression of adhesion molecule and chemokine resulted in an increase in the number of THP-1 cells attached to HUVECs as compared with the control and DMSO groups. During the early stages of atherosclerosis development, monocytes are recruited to the region of injured endothelial cells owing to the chemoattractant gradient. These cells attach to the endothelial cells via adhesion molecules such as ICAM-1 and then migrate into the vascular intima, wherein they differentiate into macrophages, internalize lipoprotein particles, and eventually become foam cells [27]. The PA-induced elevation in the expression levels of ICAM-1 and MCP-1 was prevented by the cotreatment with omentin-1; the number of THP-1 cells attached to HUVECs reduced following cotreatment with omentin-1 as compared with that observed after treatment with PA alone. These results demonstrate that omentin-1 may prevent the adhesion of monocytes to endothelial cells. The PA-induced upregulated expression of ICAM-1 and MCP-1 suggests the change in the profile of HUVECs to a proinflammatory state. Treatment with PA increased the protein expression level of NF-*κ*B and provoked the degradation of I*κ*B*α*. It is known that I*κ*B*α* binds to NF-*κ*B and covers the nuclear localization sequence of NF-*κ*B, which acts as an inhibitor of NF-*κ*B. Diverse proinflammatory stimuli could activate I*κ*B kinase complex which specifically phosphorylates I*κ*B*α*, leading to polyubiquitinated and degraded of I*κ*B*α*. Under such circumstance, NF-*κ*B could translocate into nucleus and bind target genes including *IL-1*, *IL-6*, and *TNF-α* and stimulate their transcription eventually resulting in a proinflammatory state of HUVECs [27, 28]. In the present study, the mRNA expression levels of *IL-1*, *IL-6*, and *TNF-α* were also upregulated after the exposure of HUVECs to PA alone as compared with those observed after control and DMSO groups. The upregulation in these proinflammatory factors further validated the PA-induced activation of NF-*κ*B. These inflammatory factors induce differentiation of monocytes into macrophages and accelerate the formation of foam cells [29]. The activation of NF-*κ*B and the proinflammatory signaling cascade was inhibited by the cotreatment with omentin-1. However, the exact mechanism underlying these protective effects is unknown. Previous studies have shown that omentin-1 stimulated the AMP-activated protein kinase (AMPK) signaling pathway to suppress the TNF-*α*-induced endothelial cell inflammatory state, inhibit myocardium hypertrophy, and reverse myocardial ischemic injury [12, 30, 31]. Further studies are warranted to evaluate the exact mechanism underlying the effect of omentin-1 on PA-induced endothelial cell injury. The expression level of omentin-1 is decreased in patients with CAD [32]. Increasing omentin-1 expression may prevent the onset and development of atherosclerosis and could be a potential therapeutic strategy for CAD treatment.

In conclusion, omentin-1 could rescue the PA-induced impaired proliferation and migration capabilities of HUVECs, reduce the increased number of THP-1 cells attached to PA-induced HUVECs, and inhibit the PA-induced proinflammatory state of HUVECs. These findings demonstrate the ability of omentin-1 to ameliorate PA-induced endothelial cell injury and its potential role as an antiatherosclerosis adipocytokine.

## Data Availability

The data used to support the findings of this study are included within the article.

## Supplementary Materials

Supplementary Materials The primers used in this research.

## References

[1] Gimbrone M. A., García-Cardeña G. (2016). Endothelial cell dysfunction and the pathobiology of atherosclerosis. *Circulation Research*.

[2] Khan B. V., Parthasarathy S. S., Alexander R. W., Medford R. M. (1995). Modified low density lipoprotein and its constituents augment cytokine-activated vascular cell adhesion molecule-1 gene expression in human vascular endothelial cells. *Journal of Clinical Investigation*.

[3] Morigi M., Angioletti S., Imberti B. (1998). Leukocyte-endothelial interaction is augmented by high glucose concentrations and hyperglycemia in a NF-kB-dependent fashion. *Journal of Clinical Investigation*.

[4] Stary H. C. (2000). Natural history and histological classification of atherosclerotic lesions. *Arteriosclerosis, Thrombosis, and Vascular Biology*.

[5] Mahabadi A. A., Berg M. H., Lehmann N. (2013). Association of epicardial fat with cardiovascular risk factors and incident myocardial infarction in the general population. *Journal of the American College of Cardiology*.

[6] Caserta F., Tchkonia T., Civelek V. N. (2001). Fat depot origin affects fatty acid handling in cultured rat and human preadipocytes. *American Journal of Physiology-Endocrinology and Metabolism*.

[7] Pezeshkian M., Mahtabipour M. R. (2013). Epicardial and subcutaneous adipose tissue Fatty acids profiles in diabetic and non-diabetic patients candidate for coronary artery bypass graft. *BioImpacts*.

[8] Velloso L. A., Folli F., Saad M. J. (2015). TLR4 at the crossroads of nutrients, gut microbiota, and metabolic inflammation. *Endocrine Reviews*.

[9] Zhuang W., Wang G., Li L., Lin G., Deng Z. (2013). Omega-3 polyunsaturated fatty acids reduce vascular endothelial growth factor production and suppress endothelial wound repair. *Journal of Cardiovascular Translational Research*.

[10] Iacobellis G. (2015). Local and systemic effects of the multifaceted epicardial adipose tissue depot. *Nature Reviews Endocrinology*.

[11] Watanabe T., Watanabe-Kominato K., Takahashi Y., Kojima M., Watanabe R. (2017). Adipose tissue-derived omentin-1 function and regulation. *Comprehensive Physiology*.

[12] Zhong X., Li X., Liu F., Tan H., Shang D. (2012). Omentin inhibits TNF-*α*-induced expression of adhesion molecules in endothelial cells via ERK/NF-*κ*B pathway. *Biochemical and Biophysical Research Communications*.

[13] Watanabe K., Watanabe R., Konii H. (2016). Counteractive effects of omentin-1 against atherogenesis. *Cardiovascular Research*.

[14] Xie H., Xie P.-L., Wu X.-P. (2011). Omentin-1 attenuates arterial calcification and bone loss in osteoprotegerin-deficient mice by inhibition of RANKL expression. *Cardiovascular Research*.

[15] Zhou J.-P., Tong X.-Y., Zhu L.-P. (2017). Plasma omentin-1 level as a predictor of good coronary collateral circulation. *Journal of Atherosclerosis and Thrombosis*.

[16] Wang X.-H., Dou L.-Z., Gu C., Wang X.-Q. (2014). Plasma levels of omentin-1 and visfatin in senile patients with coronary heart disease and heart failure. *Asian Pacific Journal of Tropical Medicine*.

[17] Moran A. E., Forouzanfar M. H., Roth G. A. (2014). Temporal trends in ischemic heart disease mortality in 21 world regions, 1980 to 2010. *Circulation*.

[18] Bentzon J. F., Otsuka F., Virmani R., Falk E. (2014). Mechanisms of plaque formation and rupture. *Circulation Research*.

[19] Chlopicki S. (2015). Perspectives in pharmacology of endothelium: from bench to bedside. *Pharmacological Reports*.

[20] Hansson G. K., Libby P. (2006). The immune response in atherosclerosis: a double-edged sword. *Nature Reviews Immunology*.

[21] Xue Y., Guo T., Zou L. (2018). Evodiamine attenuates P2X7-mediated inflammatory injury of human umbilical vein endothelial cells exposed to high free fatty acids. *Oxidative Medicine and Cellular Longevity*.

[22] Sawicka M., Janowska J., Chudek J. (2016). Potential beneficial effect of some adipokines positively correlated with the adipose tissue content on the cardiovascular system. *International Journal of Cardiology*.

[23] Yang R.-Z., Lee M.-J., Hu H. (2006). Identification of omentin as a novel depot-specific adipokine in human adipose tissue: possible role in modulating insulin action. *American Journal of Physiology-Endocrinology and Metabolism*.

[24] Du Y., Ji Q., Cai L. (2016). Association between omentin-1 expression in human epicardial adipose tissue and coronary atherosclerosis. *Cardiovascular Diabetology*.

[25] Witkowski M., Landmesser U., Rauch U. (2016). Tissue factor as a link between inflammation and coagulation. *Trends in Cardiovascular Medicine*.

[26] Miller J., Knorr R., Ferrone M., Houdei R., Carron C. P., Dustin M. L. (1995). Intercellular adhesion molecule-1 dimerization and its consequences for adhesion mediated by lymphocyte function associated-1. *The Journal of Experimental Medicine*.

[27] Boring L., Gosling J., Cleary M., Charo I. F. (1998). Decreased lesion formation in CCR2−/− mice reveals a role for chemokines in the initiation of atherosclerosis. *Nature*.

[28] Libby P. (2002). Inflammation in atherosclerosis. *Nature*.

[29] Collins T., Cybulsky M. I. (2001). NF-*κ*B: pivotal mediator or innocent bystander in atherogenesis?. *Journal of Clinical Investigation*.

[30] Tabas I., Bornfeldt K. E. (2016). Macrophage phenotype and function in different stages of atherosclerosis. *Circulation Research*.

[31] Matsuo K., Shibata R., Ohashi K. (2015). Omentin functions to attenuate cardiac hypertrophic response. *Journal of Molecular and Cellular Cardiology*.

[32] Kataoka Y., Shibata R., Ohashi K. (2014). Omentin prevents myocardial ischemic injury through AMP-activated protein kinase- and Akt-dependent mechanisms. *Journal of the American College of Cardiology*.

