# Supplementary material for: Omentin-1 Ameliorated Free Fatty Acid-Induced Impairment in Proliferation, Migration, and Inflammatory States of HUVECs
Source: Cardiol Res Pract. 2020 Mar 27;2020:3054379. doi: 10.1155/2020/3054379 (PMC7140148; doi:10.1155/2020/3054379)
Supplement: Supplementary Materials — The primers used in this research. [file 3054379.f1.docx]

Supplementary File

Supplementary Table 1

Primes used in the article

| human GAPDH | forward | 5’ CCAGCAAGAGCACAAGAGGAAGAG 3’ |
| --- | --- | --- |
|  | reverse | 5’ GGTCTACATGGCAACTGTGAGGAG 3’ |
| Human ICAM-1 | forward | 5’ ACGGTGCTGGTGAGGAGAGATC 3’ |
|  | reverse | 5’ CGCTGGCAGGACAAAGGTCTG 3’ |
| Human MCP-1 | forward | 5’ CAGAAGTGGGTTCAGGATT 3’ |
|  | reverse | 5’ TGGAGTGAGTGTTCAAGTC 3’ |
| Human IL-1 | forward | 5’ CTATCATGTAAGCTATGGCCCA 3’ |
|  | reverse | 5’ GCTTAAACTCAACCGTCTCTTC 3’ |
| Human IL-6 | forward | 5’ CACTGGTCTTTTGGAGTTTGAG 3’ |
|  | reverse | 5’ GGACTTTTGTACTCATCTGCAC 3’ |
| Human TNF-α | forward | 5’ TTATTTATTTACAGATGAATG 3’ |
|  | reverse | 5’ TTAGACAACTTAATCAGA 3’ |
